# AMF colonization affects allelopathic effects of *Zea mays* L. root exudates and community structure of rhizosphere bacteria

**DOI:** 10.3389/fpls.2022.1050104

**Published:** 2022-11-24

**Authors:** Junqing Ma, Yi Xie, Yisen Yang, Changliang Jing, Xiangwei You, Juan Yang, Chenyu Sun, Shengfeng Qin, Jianhua Chen, Kexin Cao, Jinghua Huang, Yiqiang Li

**Affiliations:** ^1^ Guangxi Key Laboratory of Agro-environment and Agric-products safety, College of Agriculture, Guangxi University, Nanning, Guangxi, China; ^2^ National Demonstration Center for Experimental Plant Science Education, Guangxi University, Nanning, Guangxi, China; ^3^ Tobacco Research Institute of Chinese Academy of Agricultural Sciences, Qingdao, Shandong, China

**Keywords:** AMF, root exudates, allelopathy, soil enzyme vigor, bacterial diversity

## Abstract

Arbuscular mycorrhizal fungi (AMF) widely exist in the soil ecosystem. It has been confirmed that AMF can affect the root exudates of the host, but the chain reaction effect of changes in the root exudates has not been reported much. The change of soil microorganisms and soil enzyme vigor is a direct response to the change in the soil environment. Root exudates are an important carbon source for soil microorganisms. AMF colonization affects root exudates, which is bound to have a certain impact on soil microorganisms. This manuscript measured and analyzed the changes in root exudates and allelopathic effects of root exudates of maize after AMF colonization, as well as the enzymatic vigor and bacterial diversity of maize rhizosphere soil. The results showed that after AMF colonization, the contents of 35 compounds in maize root exudates were significantly different. The root exudates of maize can inhibit the seed germination and seedling growth of recipient plants, and AMF colonization can alleviate this situation. After AMF colonization, the comprehensive allelopathy indexes of maize root exudates on the growth of radish, cucumber, lettuce, pepper, and ryegrass seedlings decreased by 60.99%, 70.19%, 80.83%, 36.26% and 57.15% respectively. The root exudates of maize inhibited the growth of the mycelia of the pathogens of soil-borne diseases, and AMF colonization can strengthen this situation. After AMF colonization, the activities of dehydrogenase, sucrase, cellulase, polyphenol oxidase and neutral protein in maize rhizosphere soil increased significantly, while the bacterial diversity decreased but the bacterial abundance increased. This research can provide a theoretical basis for AMF to improve the stubble of maize and the intercropping mode between maize and other plants, and can also provide a reference for AMF to prevent soil-borne diseases in maize.

## 1 Introduction

AMF can symbiosis with more than 80% of higher plant roots in terrestrial ecosystems to form symbionts, which is an important part of natural ecosystems (Koide and Mosse, 2004). AMF colonization can affect host rhizosphere soil enzyme vigor and soil microbial diversity (Ma et al., 2021), meanwhile, soil microorganisms are crucial to maintaining soil fertility and the sustainability of the soil ecosystem, because they play an important role in regulating organic matter decomposition and nutrient cycling (Denef et al., 2009). Root exudates play an important role in plant microbial communication, which was the key factor to maintain the vitality of the rhizosphere micro ecosystem and the important medium for material exchange and information exchange in the plant rhizosphere ecosystem (Coskun et al., 2017). Root exudates are one of the main carbon sources of soil microorganisms (De Vries et al., 2019), which can regulate the activity of plant rhizosphere microbial communities, and affect the abundance and diversity of plant rhizosphere microorganisms (Sasse et al., 2018; Jin et al., 2019).

AMF colonization can induce changes in host root exudates (Ma et al., 2022), Zhang’s research found that AMF colonization can affect the level of phenolic acids in cotton root exudates, thereby reducing the incidence rate of Cotton Fusarium Wilt (Zhang et al., 2012). After AMF colonization, it can limit the movement of nematodes and alleviate the invasion of nematodes to tomatoes by affecting the release of root exudates (Yizhu et al., 2020). AMF colonization can improve resistance by inducing changes in host root exudates. While AMF colonization affects the changes in root exudates of plants, the changes in the exudates will also affect the growth and development of AMF. They interact with each other rather than exist independently (Nagahashi and Douds, 2007).

Allelopathy mainly researches the interaction between plants & plants and between plants & microorganisms. Root exudates are one of the main ways to release allelochemicals into the environment (Li et al., 2019), which was one of the important reasons for continuous cropping obstacles (Liu et al., 2020). The Autotoxicity of root exudates (that is, root exudates from a given plant have toxic effects on themselves) has been recognized to be universal (Sun and He, 2019). Root exudates also have certain effects on the growth of other plants. Kato’s research shows that the hydroponic solution of Buckwheat Seedlings inhibits the growth of celery, lettuce, and ryegrass seedlings (Kato-Noguchi et al., 2007). However, the effects of root exudates of host plants on the growth of other plants after AMF colonization are rarely reported.

The inhibitory effect of plant root exudates on pathogenic bacteria has also been confirmed. Fu’s research shows that the root exudates of potatoes and onions can inhibit the development of tomato Verticillium Wilt (Fu et al., 2015); Li’s research shows that the change of root exudates of transgenic insect-resistant cotton leads to the reduction of its resistance to *Fusarium oxysporum* (Li et al., 2013); Turk’s research found that the hydroponic solution of black mustard can inhibit the growth of wild oats (Turk and Tawaha, 2003). At the same time, Shukla’s study found that AMF colonization can improve host resistance to Fusarium oxysporum (Shukla et al., 2015). After AMF colonization, the inhibition effect of host root exudates on pathogenic bacteria was rarely reported.

Therefore, because of the important role of AMF and root exudates in the soil ecosystem, this paper studied the changes of maize root exudates after AMF colonization, measured the effects of maize root exudates on the seed germination and seedling growth of recipient plants, and measured the changes of inhibition on the mycelium growth of soil-borne disease pathogens. At the same time, the changes in enzyme vigor and bacterial diversity in maize rhizosphere soil after AMF colonization were also measured. This research can provide the theoretical basis for AMF in maize disease prevention and intercropping, and provide research ideas for green production and sustainable development of agriculture.

## 2 Materials and methods

### 2.1 Experimental materials

A greenhouse experiment was conducted at the College of Agriculture of Guangxi University in Nanning (22°50′N, 108°17′E), China, in 2018. The maize variety Zhengda 619 (the kernel type of this kind of maize is flint corn, a common single cross hybrid that can effectively resist leaf blight, *Bipolaris maydis* and sheath blight) was provided by the Zhengda seed industry in Sichuan, China. *Claroideoglomus etunicatum* with a spore density of 71 num g^-1^ (isolated from the soil around the roots of maize plants on farmland and obtained after one year of maize propagation with Zhengda 619) was used in this experiment, the experimental container was a plastic flower pot with a 35 cm × 25 cm height and diameter. Sterilized river sand (the sand was washed with water three times to remove impurities and then sterilized by the damp heat method for 24 h.) was used as a substrate to grow the maize plants.

Radish (*Raphanus sativus* L., Cruciferae, dicotyledon; provided by Guangdong Shicheng Seed Co., Ltd.), cucumber (*Cucumis sativus* L., Cucurbitaceae, dicotyledon; provided by Fuzhou Minhang Seed Industry Co., Ltd.), lettuce (*Lactuca sativa* var. ramosa short, Compositae, dicotyledon; provided by Fuzhou Minhang Seed Industry Co., Ltd.), pepper (*Capsicum annum* L, Solanaceae, dicotyledon; provided by Fuzhou Minhang Seed Industry Co., Ltd.) and ryegrass (*Lolium perenne* L., Gramineae, monocotyledon; provided by Henan century Tianyuan Ecological Technology Co., Ltd.) was used as the recipient plant for the allelopathy test of maize root exudates.

*Fusarium graminearum* (FG), *Fusarium oxysporum* (FO) and *Rhizoctonia solani* (RS) were selected as the pathogens for the allelopathy test of maize root exudates, the above three pathogens were provided by the plant pathology research team of College of Agriculture, Guangxi University.

### 2.2 Design of the experiments

The maize seeds were washed three times with sterile water to remove impurities on the surface, soaked in 10% hydrogen peroxide for one minute to remove miscellaneous bacteria on the surface of the seeds, and then washed with sterile water. The sterilized seeds were sown on filter paper in petri dishes under control conditions at 28 °C. The evenly germinated seeds were planted in pots filled with 4 kg sterilized river sand.

Two treatments were designed: maize colonized by *Claroideoglomus etunicatum* was recorded as CE, and without AMF was recorded as CK. Put 300g inoculant containing *C.etunicum* spores into CE and put the same amount of sterilized river sand into CK for the control treatment.

Each pot was filled with 6 maize seeds that germinated. When the maize plants grew two real leaves, thinning was carried out, and one plant was retained in each pot. The filtrates were collected by mixing 300 g each of the AMF and sterilized sand together, rinsing with 5 L of sterile water, and then filtering through a 400-mesh strainer. The collected filtrates were evenly added to each pot, and 30 ml was added to each pot to ensure that the various pots had consistent microbial communities except with a difference in AMF. Each pot was irrigated with 300 ml of tap water once a week. Hogland’s nutrient solution was applied every 2 weeks from the emergence of maize plants to the 47th day and applied once a week from the 48th day to the 82nd day after emergence, applying 200 ml each time. Samples were taken at the blister stage (90 days after planting) of maize.

### 2.3 Sampling and test methods

#### 2.3.1 Collection and determination of maize root exudates

Isolation, concentration, and determination of maize root exudates were performed by reference to Ma (Ma et al., 2022). Briefly, the maize was carefully removed from the pots, cleaned of impurities on the surface of the net root system, and then transferred to a 2 L beaker after rinsing three times with sterile water. After 3 h, the culture liquid was collected, extracted, and concentrated with petroleum ether, ethyl acetate, and acetonitrile, respectively. Then the concentrated liquid obtained from the three organic phases was mixed and tested.

LC-MS was used for nontargeted determination of corn root exudates. The instrument was the UHPLC-Q Executive HF-X system. The chromatographic column is ACQUITY UPLC HSS T3 (100 mm × 2.1 mm i.d., 1.8μm; Waters, Milford, USA);Mobile phase A is 95% water + 5% acetonitrile (containing 0.1% formic acid), mobile phase B is 47.5% acetonitrile + 47.5% isopropanol + 5% water (containing 0.1% formic acid), and the injection volume is 2 μl. The column temperature is 40°C; The elution gradient of mobile phase is shown in **Supplementary Table 1**. The sample is ionized by electrospray, and the mass spectrum signals are collected by positive and negative ion scanning modes respectively. The detailed parameters of mass spectrum conditions are supplemented as shown in **Table 1**. The quality control (QC) sample is a mixture of all samples, and the volume of the QC sample is consistent with that of the sample. In the process of analysis, quality control samples shall be tested every 15 samples to verify the stability of the testing process.

**Table 1:** Difference of enzyme vigor between CE and CK in rhizosphere soil.

Soil enzyme	Original value (U/g soil)	Control	Soil enzyme vigor (U/g soil)	*p* value	Difference	Change rate (%)	Standard curve
SHDA	1.53	CE	5.19	0.0002	***	239.3	/
		CK	2.66			74.02	
SSC	152.87	CE	311.08	0.002	**	103.49	y=4.6009x+0.01
		CK	238.68			56.13	
SCL	8.78	CE	13.24	0.0004	***	50.78	y=1.78431x+0.084
		CK	9.07			3.27	
SPPO	3.43	CE	5.72	0.0296	*	66.73	y=8.2239x+0.0323
		CK	4.66			35.78	
SNEP	0.24	CE	0.38	0.0041	**	59.02	/
		CK	0.26			7.39	

SHAD, SSC, SCL, SPPO, and SNEP are the activities of dehydrogenase, sucrase, cellulase, polyphenol oxidase, and neutral protease in soil respectively. * represents significant difference between CE and CK (p<0.05), * * represents extremely significant difference between CE and CK (p< 0.01), *** represents extremely significant difference between CE and CK (p<0.001).

Import the original data into the metabonomics processing software ProgenesisQI (Waters Corporation, Milford, USA) for baseline filtering, peak identification, integration, retention time correction, peak alignment, and finally obtain a data matrix containing information such as retention time, mass charge ratio, and peak intensity. Then the software was used to identify the characteristic peak database, and the MS and MS/MS mass spectrum information was matched with the metabolic database. The MS mass error was set to be less than 10 ppm. At the same time, the metabolites were identified according to the matching score of the secondary mass spectrum. The substances with unique mass charge ratio, unique retention time and theoretical fragment fraction ≥ 50 are considered as effective compounds determined this time, which are listed in HMDB Version 5.0 through the Library ID of the compounds (https://Human.Metabolome.Database (hmdb. Ca), classify the detected substances according to the search results, and perform principal component analysis on the detected compounds according to their relative content.

#### 2.3.2 Effect of maize root exudates on the growth of recipient plants

Accurately suck 2 ml of the collected root exudate concentrate and evenly coat it in the seed germination bag. The germination bag coated with CE root exudates is marked as CE, and the germination bag coated with CK root exudates is marked as CK; Since the root exudates of this test are finally eluted with chromatographic methanol, a seed germination bag with only 2 ml of chromatographic pure methanol is set as blank processing, and it is recorded as BP. Put the treated seed germination bag into the fume hood, blow until the methanol is completely volatilized, add 2 ml of sterilized ultrapure water, place healthy and full recipient plant seeds in each seed culture bag, and set 6 repetitions for each treatment.

The whole seed culture was transferred to an artificial intelligence incubator (LRH-400-G, Taihong Medical Instrument Co., Ltd., Shaoguan, China) for constant temperature culture at 25°C. Each day was incubated in light for 12 h, dark for 12 h, and 2 ml sterilized ultrapure water was added every 24 h. The number of newly germinated seeds and total germinated seeds were counted daily for 7 consecutive days since being transferred to the incubator. The rates of Percentage of Seed germination (GP), Germination index (GI), and Germination energy (GE) in the recipient plants were calculated according to Shamya (Shamya Arokia Rajan et al., 2020).

#### 2.3.3 Effect of maize root exudates on the growth of pathogenic fungi

Root exudate concentrate of CE and CK were respectively configured into a bacteriostatic solution of 0, 1, 2, 3, 4, and 5 mg/ml for use. The inhibitory effect of maize root exudates on the mycelial growth of *Fusarium graminearum*, *Fusarium oxysporum*, and *Rhizoctonia solani* was determined using the mycelial growth method (Rabea et al., 2009). 15 ml of sterilized PDA medium was poured into a sterile Petri dish and allowed to solidify. Place the four oxford cups on the surface of the PDA medium and press gently to make the cup and agar surface free of voids, place 200 μL root exudate extract injected into the cup and spread for 24h.

According to the perforated hole method (Adedokun et al., 2016), the clots of *Fusarium graminearum*, *Fusarium oxysporum*, and *Rhizoctonia solani* revived for one week were filled into the pretreated medium by individually hitting a hole in the center of the medium with sterilized gun heads and transferred to a 28°C constant temperature electrothermal incubator (DNP-9082, sperm along experimental equipment Co., Ltd., Shanghai) for 5 days. The mycelial diameter of pathogenic fungi was measured, and the fungal inhibition effect was calculated according to Rabea (Rabea et al., 2009).

#### 2.3.4 Determination of rhizosphere microorganisms

Soil total genomic DNA was extracted using the Mn Nucleospin 96 Soi Kit (MACHEREY-NAGEL, Dueren, Germany) according to the guide method of the kit.

The bacterial 16S V3 + V4 region was PCR amplified using a thermal cycler PCR system (gene amp 9700, ABI, USA) with primer pairs 338F (5′-ACTCCTACGGGAGGCAGCA-3′) and 806R (5′-GGACTACHVGGGTWTCTAAT-3′) (Chen et al., 2018). This assay uses phusion high fidelity polymerase (TOYOBO KOD FX Neo, multiplate, Japan) to extract PCR products from bacteria on a 1.8% agarose gel.

PCR amplification was performed according to the method of Muhammad (Muhammad et al., 2022). The PCR reaction voltage was 120V, and the reaction time was 40 min; The initial denaturation at 98°C for 30s was followed by 10 cycles (denaturation at 98°C for 10s, annealing at 65°C for 30s, and extension at 72°C for 30s), and a final extension at 72°C for 5 min. Purified using OMEGA DNA Purification Kit (Omega Inc., Norcross Georgia, USA) followed by recovery using Monarch DNA gel Recovery Kit (Lumiprobe Inc., Rutgers Maryland, USA).

The PCR products were purified, pooled in equimolar amounts, then subjected to high-throughput sequcencing with an Illumina Hiseq 2500 platform (Illumina, San Diego, CA, United States) (Yang et al., 2022). Raw data were quality filtered using Trimmomatic V 0.33 (Magoc and Salzberg, 2011) before primer sequence identification and removal using Cutadapt v1.9.1 (Bolger et al., 2014); High quality reads from each sample were then tiled by overlap using Flash v1.2.11 to yield Clean Reads (Edgar et al., 2011), and finally, using uchime v8.1, chimera sequences were identified and removed to yield final effective reads (Edgar, 2013)., Selected Silva database (http://www.arb-silva.de)to alignment the assay results (Quast et al., 2012). Using RDP classifier V2.2(http://sourceforge.net/projects/rdpclassifier/)Species annotation of bacteria in the phylum, order, order, family, genus, and species was performed with a confidence threshold of 0.8 (Wang et al., 2007). Bacterial phylogenetic tree analysis of the multiple alignment database was PyNAST v1.2.2 (http://biocore.github.io/pynast/) (Caporaso et al., 2010).

### 2.4 Statistical analysis

Statistical analysis was conducted using Microsoft Excel 2013, SPSS (20.0, IBM, USA), and Origin 2021 (OriginLab Corp., Northampton, MA, USA). One-way analysis of variance (ANOVA) with Duncan tests was used to compare the differences between treatments ≤ 0.05.

Principal component analysis of the detected active substances was performed by simca14.1, the data were subjected to 200 hypothesis tests using permutation module, then VIP predictive was used to obtain variable VIP values of compounds in CE and CK root exudates, and the compounds with VIP > 1 were those with significant differences in CE and CK root exudates.

## 3 Results

### 3.1 Root exudate changes in maize after AMF colonization

**Figure 1:**
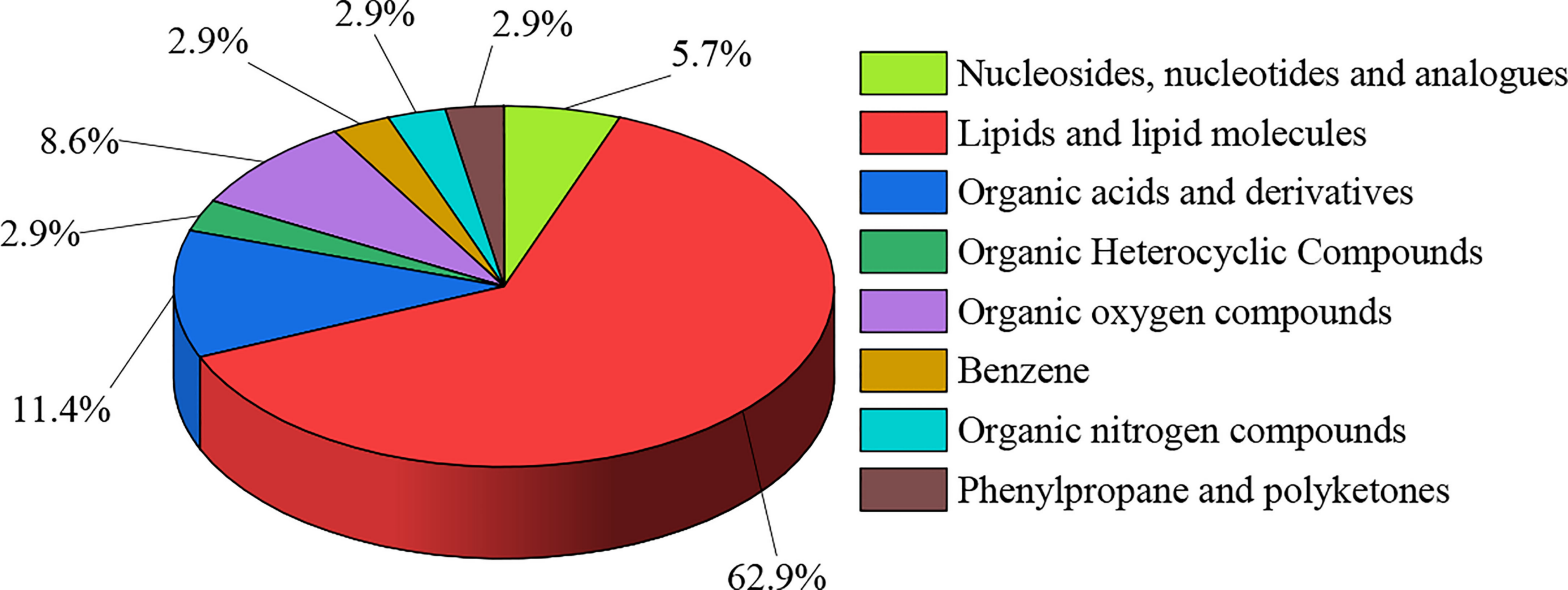
Classification map of differential compounds in maize root exudates after AMF colonization.

**Figure 2:**
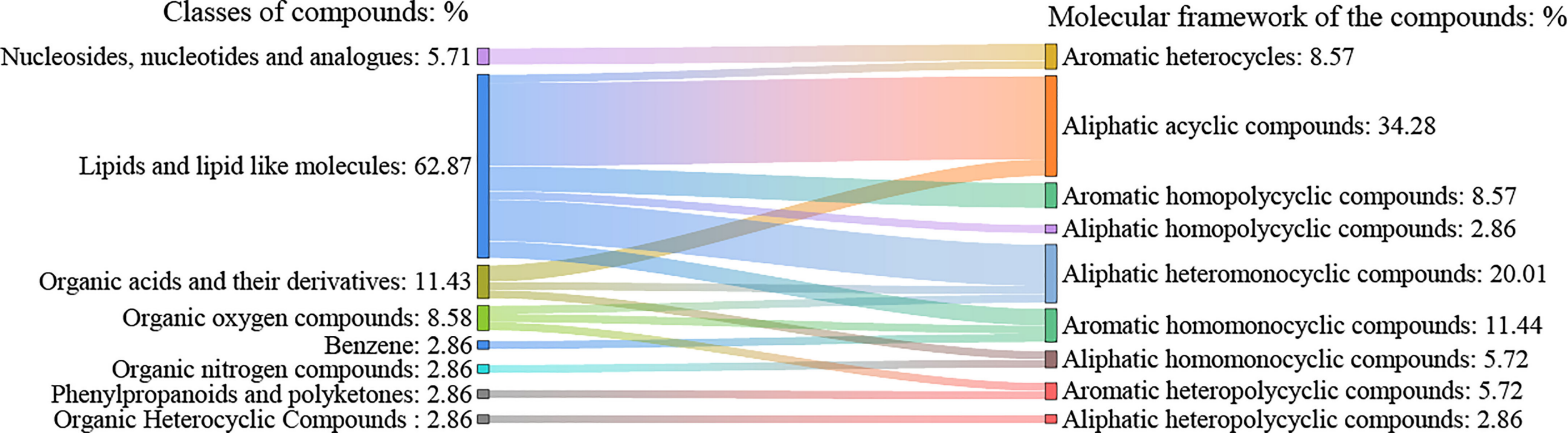
Molecular framework statistical map of differential compounds in maize root exudates after AMF colonization. The left side of the figure shows the classification of maize root exudates with significant differences after AMF colonization, and the right side shows the carbon skeleton corresponding to these root exudates.

A total of 412 active compounds were detected in this LC-MS assay from maize root exudates, 262 of which were detected under cationic conditions and 150 under anionic conditions. Yielding a total of 35 key compounds with significant differences. The 35 key compounds that were significantly different in maize root exudates after *C. etunicatum* colonization were subjected to taxonomic statistical analysis. The results showed that it mainly included 2 kinds of nucleosides, nucleotides, and analogs, accounting for 5.71%; 22 lipid and lipid-like molecules, 62.86%; 4 kinds of organic acids and their derivatives, accounting for 11.43%; 1, organic heterocycles, accounting for 2.86%; 3 kinds of organic oxygen compounds, accounting for 8.57%; 1 for benzene, 2.86%; 1 organic nitrogen compounds, accounting for 2.86%; 1 of phenylpropanoid and polyketide, 2.86%. The differential compound classification plot is shown in **Figure 1**. Statistical analysis of the molecular frameworks of the above compounds showed that the molecular frameworks of the differential compounds in maize root exudates after AMF colonization were mainly aromatic acyclic compounds accounting for 34.28%, followed by aliphatic heteromonocyclic compounds accounting for 20.01%, and the results are shown in **Figure 2**.

### 3.2 Effect of root exudates on the growth of recipient plants

#### 3.2.1 Effect of root exudates on seed germination in recipient plants

**Figure 3:**
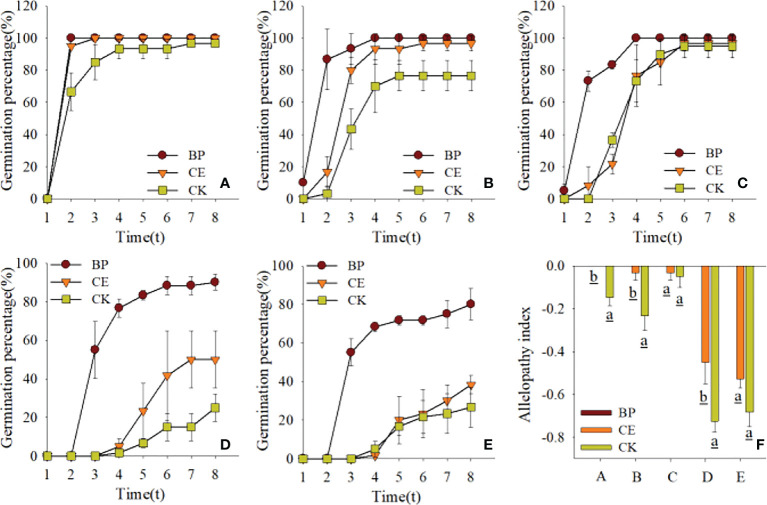
Effect of CE and CK root exudates on seed germination rate in recipient plants. **(A–E)** shows the effects of CE and CK root exudates on the seed germination rate of radish, cucumber, lettuce, pepper, and ryegrass respectively, and **(F)** shows the difference of allelopathic effects of CE and CK root exudates on the germination rate of the above receptor plants; **(A–E)** represent radish, cucumber, lettuce, pepper, and ryegrass respectively.

The effects of CE and CK root exudates on seed germination rates and chemosensory effects in the recipient plants are shown in **Figure 3**. Overall, the germination rate of radish seeds was comparatively less affected by maize root exudates, and that of ryegrass seeds was comparatively more affected by maize root exudates. The germination rate of seeds from each recipient plant was stable on the eighth day of the germination experiment, and the cucumber seed germination rates under the action of BP, CE, and CK root exudates were 100%, 96.67%, and 76.67%, respectively; The seed germination rates were 90%, 50%, and 25% for pepper and 81.67%, 38.33%, and 26.67% for ryegrass under the same treatments, respectively. We could find such a rule that the germination rates of cucumber seeds, pepper seeds, and ryegrass seeds under the action of CE and CK root exudates were all inhibited compared with BP, while the inhibitory effect of CK root exudates on seed germination rates mentioned above was especially obvious. The findings illustrate that maize root exudates act to suppress the germination of recipient plants, whereas AMF colonization is followed by a reduction in the inhibitory effect of maize root exudates on Seed Germination of recipient plants.

**Figure 4:**
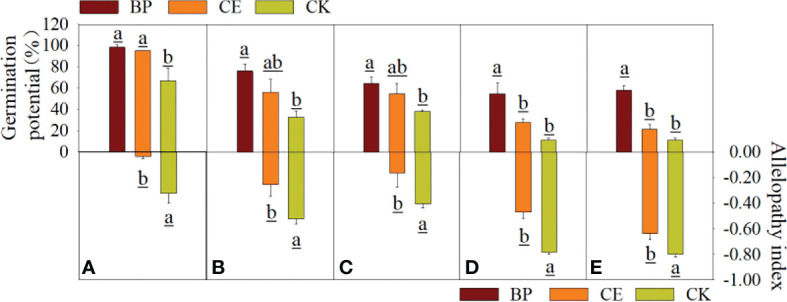
Effect of CE and CK root exudates on seed germination potential of recipient plants. **(A–E)** shows the effects of CE and CK root exudates on seed germination potential and allelopathic effect index of radish, cucumber, lettuce, pepper, and ryegrass, respectively.

The effects of CE and CK root exudates on seed germination potential and chemosensory effects in the recipient plants are shown in **Figure 4**. Although radish seed germination was less affected by root exudates than maize, its germination potential was more affected by root exudates. As can be seen from **Figure 4**, the germination potential of seeds of each recipient plant under the action of maize root exudates was significantly affected, especially the germination potential of seeds of each recipient plant under the action of CK root exudates was significantly lower than that of BP (*p* < 0.05). The germination potential of seeds from pepper and ryegrass was particularly affected by maize root exudates and was significantly lower (*p* < 0.05) for both CE and CK root exudates than for BP. As can be seen from **Figure 4**, the chemosensory effect index of CE root exudates on seed germination potential of each recipient plant was significantly lower than that of CK, indicating that AMF post-colonization would reduce the inhibitory effect of maize root exudates on seed germination potential of the recipient plant.

**Figure 5:**
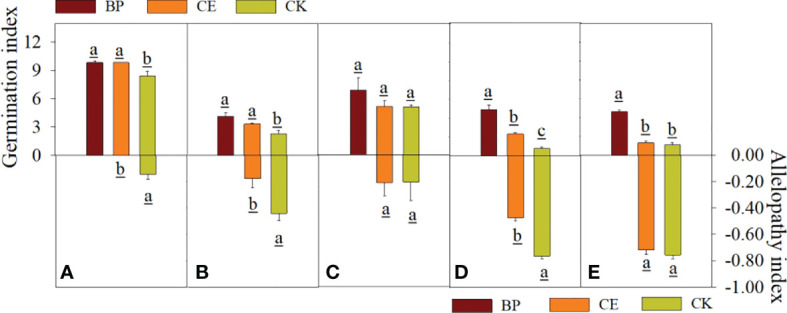
Effect of CE and CK root exudates on seed germination index in recipient plants. **(A–E)** shows the effects of CE and CK root exudates on seed germination index and allelopathic effect index of radish, cucumber, lettuce, pepper, and ryegrass respectively.

The effects of CE and CK root exudates on seed germination index and Allelopathy of recipient plants are shown in **Figure 5**. The germination index of capsicum and ryegrass seeds was significantly decreased by maize root exudates (*p* < 0.05), and the inhibition effect of CK root exudates on the germination index of Capsicum seeds was significantly higher than that of CE (*p* < 0.05). The allelopathic effect index of CE root exudates on the germination index of cucumber, pepper, and ryegrass seeds was significantly lower than CK, indicating that maize root exudates can reduce the germination index of recipient plant seeds, and AMF colonization can alleviate this effect.

#### 3.2.2 Effect of root exudates on seedling growth of recipient plants

**Figure 6:**
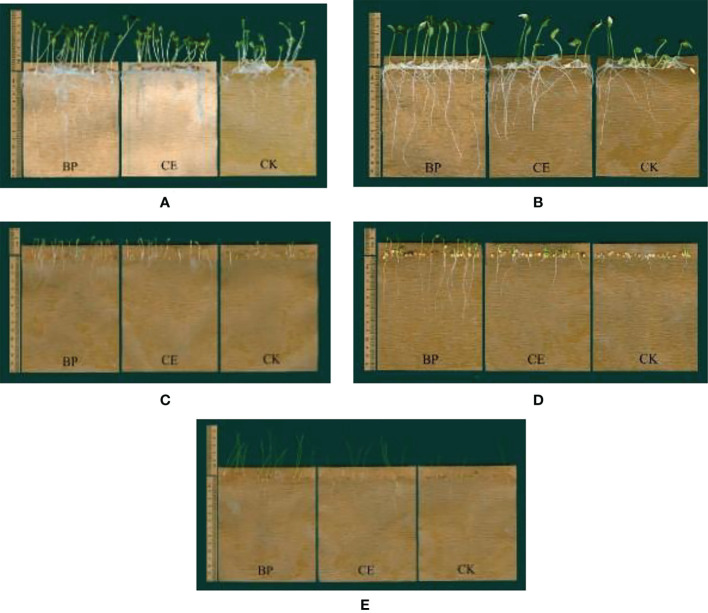
Effect of maize root exudates on the growth of recipient seedlings. **(A–E)** shows the effects of CE and CK root exudates on the growth of radish, cucumber, lettuce, pepper and ryegrass respectively. BP indicates the blank control without any treatment, CE indicates the effect of maize root exudates on recipient plant seedling growth after *C.etunicum* colonization, CK indicates the effect of maize root exudates without AMF colonization on recipient plant seedling growth.

It can be seen from **Figure 6** that the root exudates of maize inhibit the growth of recipient plant seedlings, especially the growth of cucumber, lettuce, pepper, and ryegrass seedlings. The inhibitory effect of CE root exudates on the growth of recipient plants is less than CK, which indicates that AMF colonization reduces the inhibitory effect of maize root exudates on the growth of recipient plants.

The hypocotyl length of recipient plants was inhibited by maize root exudates to varying degrees. The hypocotyl length of each recipient under the action of CK root exudates was significantly lower than BP (*p* < 0.05), and the hypocotyl length of lettuce and pepper under the action of CE root exudates was lower than BP, but the difference was not significant. Except for radish, the radicle length of other recipient plants was significantly inhibited by maize root exudates, and CE root exudates significantly reduced the inhibitory effect of maize root exudates on the radicle length of lettuce (*p* < 0.05). The fresh weight and dry matter accumulation of radicle and Hypocotyl of each recipient plant were significantly inhibited by root exudates of maize. CE root exudates significantly reduced the inhibition of maize root exudates on fresh weight of cucumber and lettuce hypocotyls (*p* < 0.05), decreased the inhibition of maize root exudates on dry matter accumulation of lettuce and ryegrass hypocotyls (*p* < 0.05), and decreased the inhibition of maize root exudates on dry matter accumulation of radish Hypocotyls (*p* < 0.05). The seedling growth, fresh weight, and dry matter accumulation of each recipient plant under the action of maize root exudates are shown in **Supplementary Table 2**.

**Figure 7:**
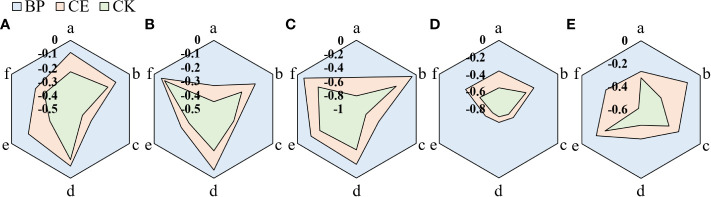
Allelopathic effect index of root exudates of Maize on the growth of seedlings of recipient plants. **(A–E)** shows the allelopathic effect index of CE and CK root exudates on the growth of radish, cucumber, lettuce, pepper, and ryegrass respectively. (a–f) in the figure respectively represent the allelopathic effect index of maize root exudates on the hypocotyl length, bacon length, fresh weight of hypocotyl, fresh weight of mating root, dry matter accumulation of hypocotyl, and dry matter accumulation of radicle of the recipient plant.

The allelopathic effect of maize root exudates on the growth of recipient plant seedlings is shown in **Figure 7**. Maize root exudates inhibit the growth of recipient plant seedlings, but after AMF colonization, the inhibitory effect of root exudates on the growth of recipient plant seedlings is reduced.

### 3.3 Effect of root exudates on mycelial growth of soil-borne diseases

**Figure 8:**
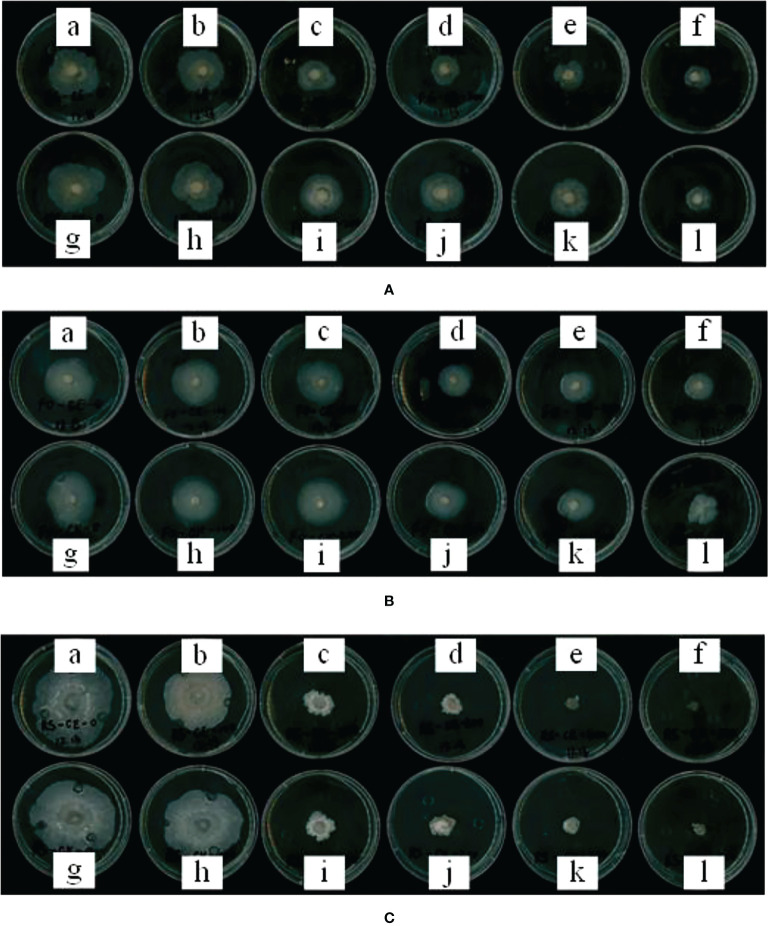
Effect of root exudates of CE and CK on mycelial growth of soil-borne diseases. **(A–C)** shows the effect of root exudates on the mycelial growth of *Fusarium graminearum*, *Fusarium oxysporum* and *Rhizoctonia solani*, respectively. (a–f) in each figure shows the mycelial growth of mycelium after adding CE root exudates with concentrations of 0, 1, 2, 3, 4, and 5 mg/ml for 5 days, and figures (g–l) shows the mycelial growth of mycelium after adding CK root exudates with concentrations of 0, 1, 2, 3, 4 and 5 mg/ml for 5 days; 0 mg/ml is the blank control (BP).

The effect of maize root exudates on the mycelial growth of *Fusarium graminearum*, *Fusarium oxysporum* and *Rhizoctonia solani* is shown in **Figure 8**. With the increase in the concentration of root exudates, the inhibition effect of root exudates on the mycelial growth of pathogenic bacteria increased, and the inhibition effect of maize root exudates on the mycelial growth of *Rhizoctonia solani* was the strongest.

When the concentration of maize root exudates was 2 mg/ml, the growth of *Fusarium graminearum* mycelium was inhibited. At this concentration, the inhibitory rates of CE and CK root exudates against *Fusarium graminearum* were 36.37% and 21.53%, respectively. The inhibitory effect of CE root exudates on the growth of *Fusarium graminearum* mycelium was significantly higher than CK (*p* < 0.01). When the concentration of root exudates was 5 mg/ml, the mycelial growth diameter of *Fusarium graminearum* under the action of CE and CK root exudates was 17.37 mm and 19.09 mm respectively, and the mycelial growth diameter was less than half of that of BP. The inhibition rates of CE and CK root exudates against *Fusarium graminearum* were 54.54% and 53.42% respectively.

When the concentration of maize root exudates was 3 mg/ml, the growth of *Fusarium oxysporum* mycelium was inhibited. The inhibition rates of CE and CK root exudates to *Fusarium oxysporum* mycelium were 35.89% and 24.70%, respectively. The inhibition effect of CE root exudates on the growth of *Fusarium oxysporum* mycelium was significantly higher than CK (*p* < 0.01). With the increase in the concentration of maize root exudates, its inhibitory effect on the mycelial growth of *Fusarium oxysporum* was further strengthened. When the concentration of maize root exudates was 5 mg/ml, the growth diameter of the mycelial growth of *Fusarium oxysporum* under the action of CE and CK root exudates was 20.36 mm and 22.34 mm respectively, and the inhibitory rate of CE and CK root exudates against *Fusarium oxysporum* was 45.64% and 40.40% respectively. The inhibitory effect of CE root exudates on the mycelial growth of *Fusarium oxysporum* was significantly higher than CK (*p* < 0.05).

When the concentration of maize root exudates was 1 mg/ml, the growth of *Rhizoctonia solani* was inhibited. At this concentration, the inhibition rates of CE and CK root exudates against *Rhizoctonia solani* were 15.48% and 10.12%, respectively. The inhibition effect of CE root exudates on the growth of *Rhizoctonia solani* was significantly higher than CK (*p* < 0.01). With the increase in the concentration of root exudates, the bacteriostatic rate of CE and CK against *Rhizoctonia solani* increased step by step. When the concentration of root exudates was 4 mg/ml, the bacteriostatic rate of CE and CK against *Rhizoctonia solani* reached more than 80%. At this concentration, the bacteriostatic rate of CE and CK against *Rhizoctonia solani* was 85.18% and 80.03% respectively. Under the same concentration, the inhibitory effect of CE root exudates on the mycelial growth of *Rhizoctonia solani* was significantly higher than CK (*p* < 0.01).

The inhibitory effects of different treatments on the mycelial growth of pathogenic bacteria are shown in **Supplementary Table 3**.

### 3.4 Difference of enzyme vigor between CE and CK in rhizosphere soil

After AMF colonization, the soil enzyme vigor in the maize rhizosphere soil generally increased. The soil enzyme vigor in the maize rhizosphere soil after AMF colonization is shown in **Table 1**. The dehydrogenase activity in CE and CK rhizosphere soil increased by 3.66 and 1.13 U g^-1^ soil, respectively. After AMF colonization, the dehydrogenase activity in maize rhizosphere soil increased by 165.28% compared with CK (*p* < 0.001). The activities of sucrase in maize rhizosphere soil increased by 158.21 and 85.81 U g^-1^ soil respectively. After AMF colonization, the activities of sucrase in maize rhizosphere soil increased by 47.36% compared with CK (*p* < 0.01). The cellulase activity in maize rhizosphere soil increased by 4.46 and 0.29 U g^-1^ soil, respectively. After AMF colonization, the cellulase activity in maize rhizosphere soil increased by 47.51% compared with CK, and the difference was significant (*p* < 0.001). Polyphenol oxidase activity increased by 2.29 and 1.23 U g^-1^ soil, respectively. After AMF colonization, polyphenol oxidase activity in maize rhizosphere soil increased by 30.95% compared with CK, and the difference was significant (*p* < 0.05). Neutral protease activity increased by 0.38 and 0.26 U g^-1^ soil, respectively. After AMF colonization, neutral protease activity in maize rhizosphere soil increased by 51.36% compared with CK, and the difference was significant (*p* < 0.01).

### 3.5 Effects of AMF colonization on soil bacteria in maize rhizosphere

Alpha–diversity of the rhizosphere soil of maize is shows in **Table 2**, after quality filtering, 1888 OTUs and 1869 OTUs were identified from the rhizosphere soil microbial RNA sequences of CE and CK, respectively. The results showed that the bacterial abundance (ACE and Chao1) of CE rhizosphere soil was significantly higher than CK (*p*< 0.05), but the bacterial diversity (Simpson and Shannon) was significantly lower than CK (*p* < 0.05).

**Table 2:** Alpha–diversity of the rhizosphere soil of Maize.

Sample ID	ACE	Chao1	Simpson	Shannon
CE	1856.65 ± 0.70a	1868.14 ± 1.94a	0.99 ± 0b	9.12 ± 0.02b
CK	1836.14 ± 7.12b	1846.43 ± 6.17b	1.00 ± 0a	9.40 ± 0.02a

**Figure 9:**
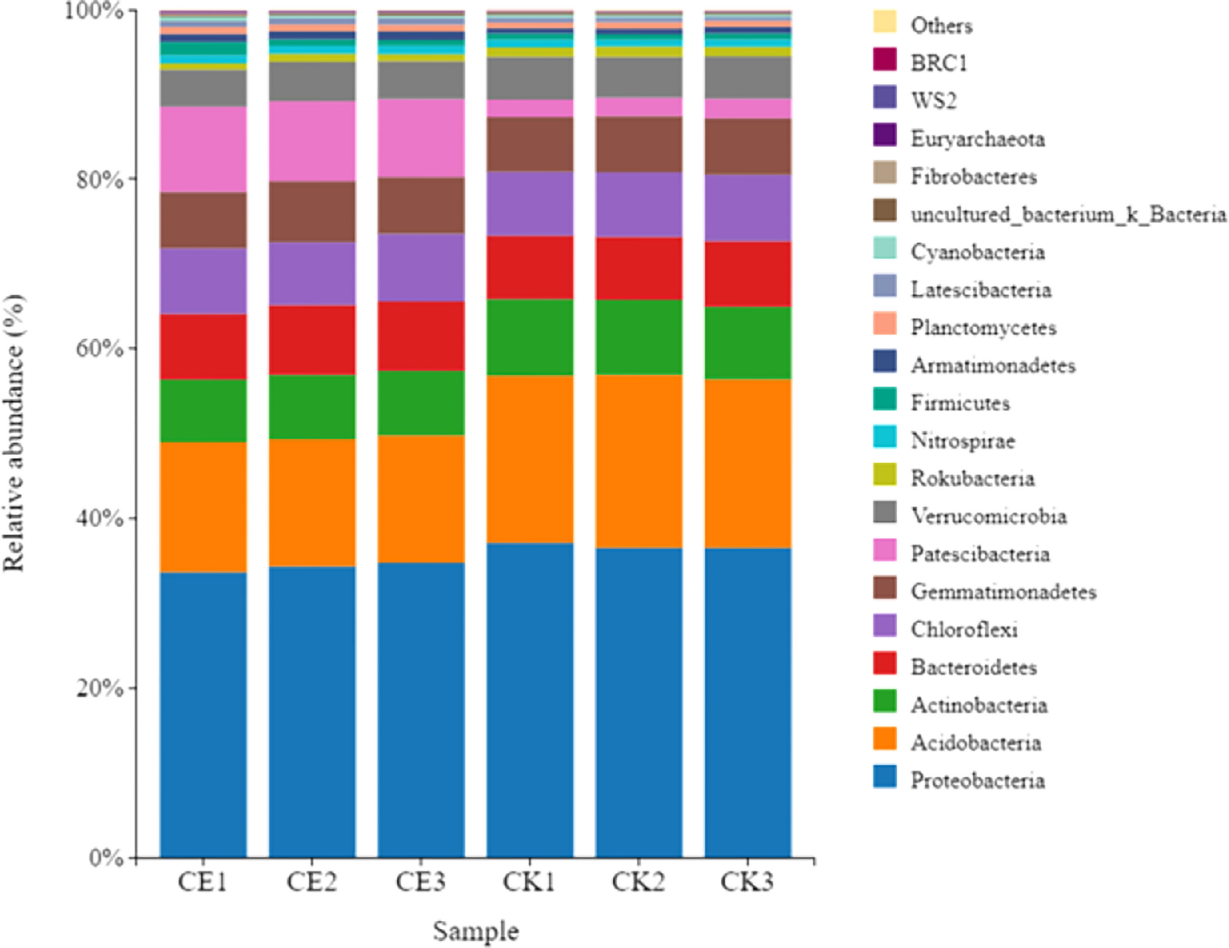
Differences in bacterial phylum level distribution in maize rhizosphere soils.

The results of analysis on the distribution difference of bacteria in maize rhizosphere soil at phylum level showed that Proteobacteria was the most abundant bacteria in CE and CK rhizosphere soil, accounting for 34.24% and 36.71% respectively; The bacteria with the greatest difference in abundance were paesciabacteria, accounting for 9.56% and 2.19% respectively. After AMF colonization, the abundance of paesciabacteria in maize rhizosphere soil increased by 337.10%. In addition, AMF colonization increased the abundance of Bacteroidetes, Chloroflexi, gemmatimonaetes, and nitrospirae in the rhizosphere of maize, and decreased the abundance of acidobacteria, Actinobacteria, verrucomicrobia, and rokubacteria (**Figure 9**).

**Figure 10:**
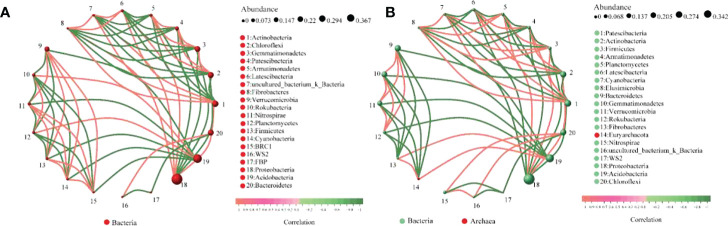
Interaction of bacteria in maize rhizosphere soil. **(A)** shows the interaction between bacteria in CE rhizosphere soil and **(B)** shows the interaction between bacteria in CK rhizosphere soil.

The interaction analysis of bacteria (top20) at the phylum level in maize rhizosphere soil is shown in **Figure 10**. The analysis results show that there are 126 pairs of interactions between CE rhizosphere soil bacteria and 134 pairs of interactions between CK rhizosphere soil bacteria; The interaction score between CE rhizosphere soil bacteria was 93.78, and that between CK rhizosphere soil bacteria was 97.98. AMF colonization inhibited the interaction between soil bacteria in maize rhizosphere.

## 4 Discussion

### 4.1 AMF colonization affected the content of each component of maize root exudates

Root exudates can improve the status of soil nutrients and improve the efficiency of nutrient absorption and utilization by plants (Nardi et al., 2002), AMF colonization can improve the ability of plants to absorb nutrients by affecting the root exudates of plants (Fusconi, 2014). AMF colonization can alleviate the continuous cropping obstacle of American ginseng, which is related to the change of root exudates (Liu et al., 2020). Tian’s research found that the increase of flavonoid concentration in root exudates can enhance the connection between arbuscular mycorrhizal fungi and host plants (Tian et al., 2021), it was found that AMF colonization significantly changed the content of various components of root exudates of maize in this study. Therefore, because of this phenomenon, this project further studied the Allelopathy of maize root exudates after AMF colonization.

### 4.2 AMF colonization alleviated the inhibition of maize root exudates on seed germination of recipient plants

The potential role of plant root exudates has been verified by many researchers (Baetz and Martinoia, 2014), which includes the effect of root exudates on plants themselves (Li et al., 2019), on other plants (Dong et al., 2022), on microorganisms and even on soil ecosystems (Tian et al., 2021). In this study, it was found that maize root exudates inhibited the seed germination of radish, cucumber, lettuce, pepper, and ryegrass, and AMF colonization alleviated the inhibition of maize root exudates on the seed germination of the above recipient plants. The germination rate of seeds is affected by many factors, such as germination temperature, humidity, biological or abiotic stress, and exogenous hormones (Song et al., 2019; Klupczyńska and Pawłowski, 2021). AMF colonization alleviates the inhibitory effect of host root exudates on the seed germination of recipient plants, which will provide a theoretical basis for the study of maize stubble modification and rotation mode with other plants.

### 4.3 AMF colonization alleviated the inhibition of maize root exudates on the growth of recipient plants

Environmental factors can directly affect the growth of plant seedlings (Sela et al., 2020), and endogenous hormones, salt content, and root exudates can also affect the growth of plant seedlings to varying degrees (Zou et al., 2019; López-Ruiz et al., 2020; Pantigoso et al., 2020). This study found that maize root exudates could inhibit the growth of radish, cucumber, lettuce, pepper, and ryegrass seedlings, and the inhibition of root exudates on the growth of radicle of recipient plants was higher than that on the growth of hypocotyl, which may be related to the direct contact between hypocotyl and root exudates of maize. After AMF colonization, the allelopathic effect index of maize root exudates on the growth of recipient plants’ seedlings was reduced, which indicates that AMF colonization alleviated the inhibitory effect of maize root exudates on the growth of recipient plants. The results of this study can provide a theoretical basis for the study of maize stubble modification and Intercropping mode with other plants.

### 4.4 AMF colonization enhanced the inhibitory effect of root exudates on the growth of pathogenic fungi

*Fusarium graminearum* can cause root rot in maize (Zhang et al., 2016), *Fusarium oxysporum* can cause stem rot in maize (Oghenekaro et al., 2021), *Rhizoctonia solani* can cause sheath blight of maize (Singh et al., 2020), and the above three pathogens are all soil-borne diseases, which seriously restrict the production of maize. Some research showed that chitosan could enhance the resistance of plants to *Fusarium oxysporum* by inducing changes in plant root exudates (Ntushelo et al., 2019; Suarez-Fernandez et al., 2020), Lanoue’s research showed that plant root exudates had a good inhibitory effect on Fusarium (Lanoue et al., 2010). This study found that maize root exudates inhibited the growth of *Fusarium graminearum*, *Fusarium oxysporum*, *Rhizoctonia solani*, and AMF colonization enhanced the inhibition of maize root exudates on the growth of pathogenic bacteria. This is consistent with Hao’s results, which show that root exudates of watermelon and rice have a good inhibitory effect on *Fusarium oxysporum* (Hao et al., 2010). The results of this study can provide a theoretical basis for AMF to study the control of plant soil-borne diseases.

### 4.5 AMF colonization affected the distribution of bacteria in maize rhizosphere soil and increased the enzyme vigor in soil

**Figure 11:**
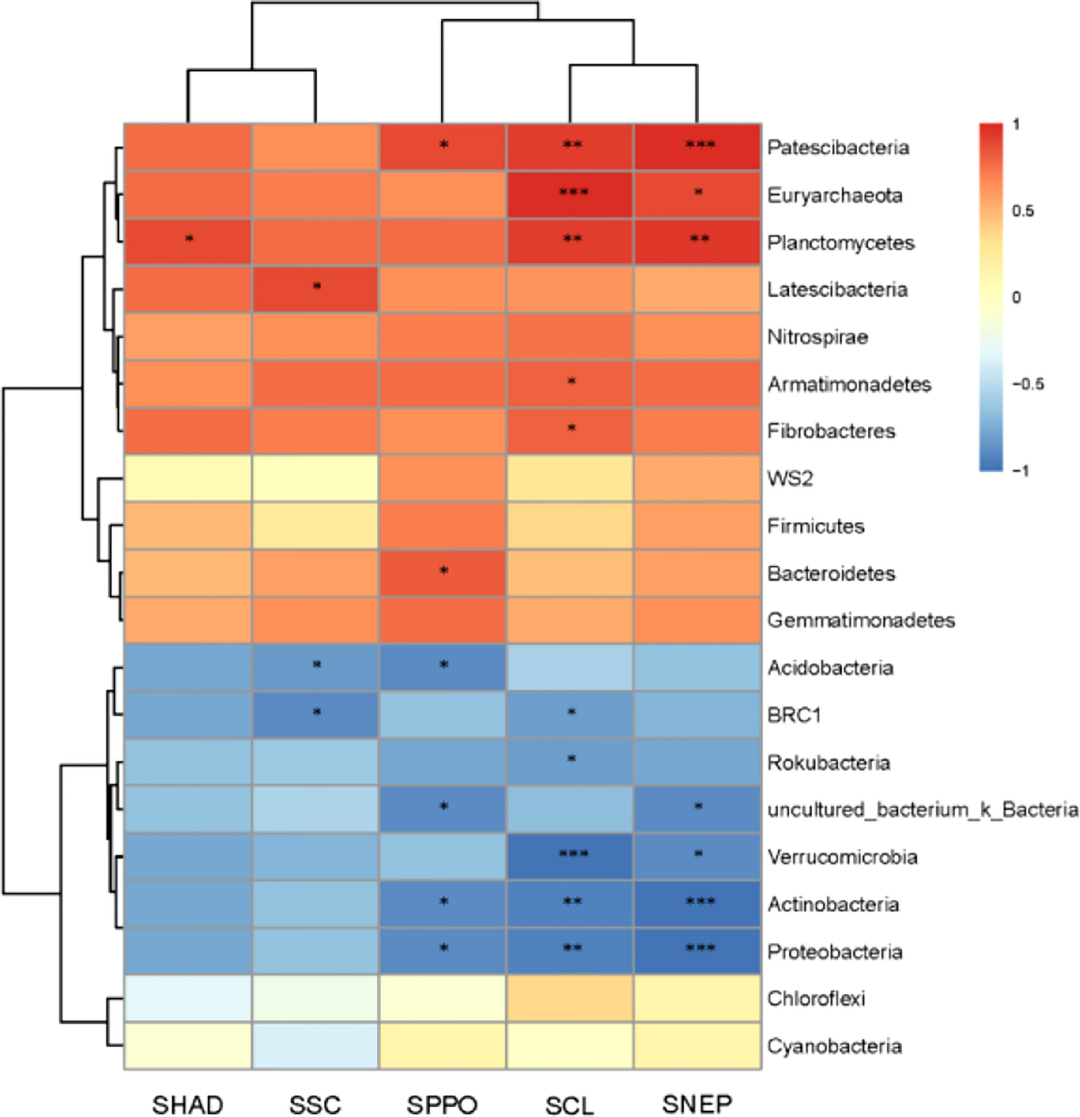
Pearson correlation between soil bacteria and soil enzyme vigor in maize root system. SHAD, SSC, SCL, SPPO, and SNEP are the activities of dehydrogenase, sucrase, cellulase, polyphenol oxidase, and neutral protease in soil respectively * represents significant difference between CE and CK (p<0.05), ** represents extremely significant difference between CE and CK (p< 0.01), *** represents extremelysignificant difference between CE and CK (p<0.001).

Soil enzyme vigor is closely related to the function of the soil ecosystem and can reflect the metabolic capacity of soil microorganisms (Zarea et al., 2011; Bowles et al., 2014), and microbial changes can directly affect the activity of soil enzymes (Pereira et al., 2016). Some studies have shown that AMF can improve the host’s absorption of mineral elements by affecting the soil enzyme vigor (Peng et al., 2020), and AMF can improve the activity of N-related enzymes in the host’s rhizosphere soil at the early stage of colonization to promote the host’s absorption of nitrogen (Stamou et al., 2017). There is also a certain correlation between the change of soil enzyme vigor and the change of root exudates. Staszel’s research shows that the root exudates of plants will decrease under drought stress, which will lead to the change of soil enzyme vigor in the rhizosphere. (Staszel et al., 2022). This study found that after AMF colonization, the activities of dehydrogenase, sucrase, cellulase, polyphenol oxidase and neutral protease in maize rhizosphere soil were significantly increased. It is speculated that the changes in enzymatic vigor in maize rhizosphere soil after AMF colonization are related to the changes in rhizosphere soil microorganisms. Therefore, the pearson correlation of bacteria and soil enzyme vigor in the rhizosphere soil of maize after AMF colonization were analyzed, the results are shown in **Figure 11**. The analysis results show that the changes of Fibrobacteres, Armimonadetes, Bacteroidetes, Euryarchaeota, Latescibacteria, Patescibacteria, and Plantomycetes are positively correlated with the changes in soil enzyme vigor in maize rhizosphere; the change of Acidobacteria, Actinobacteria, tbrc1, Proteobacteria, rokubacteria, verrucomicrobia, and uncultured_bacterium_k_ was negatively correlated with the change of enzyme vigor in maize rhizosphere soil. The effects of cellulase, polyphenol oxidase, and neutral protease on the bacterial abundance in maize rhizosphere were particularly obvious.

Soil enzyme vigor is also used as an indicator of changes in soil quality and productivity, which is mediated by microorganisms (Zhang et al., 2019). The soil microbial community is affected by many factors. Zubek’s research found that plants can affect soil microorganisms through specific root exudates (Zubek et al., 2012), therefore, AMF colonization affects host rhizosphere microbial changes by inducing host root exudates to change (Mar Vázquez et al., 2000; Cosme and Wurst, 2013). AMF interacts with soil microorganisms, AMF can affect the community composition of soil microorganisms, and the changes in soil microorganisms can also affect the reproduction of AMF (Caravaca and Ruess, 2014). This research found that AMF colonization increased the abundance of bacteria in maize rhizosphere soil and decreased the diversity of bacteria in maize rhizosphere soil. That is to say, AMF specifically screened the bacteria in the maize rhizosphere soil, optimized the level of the bacteria in the maize rhizosphere soil, increased the number of functional bacteria in the maize rhizosphere soil, and improved the enzyme vigor of the rhizosphere soil.

## 5 Conclusions

AMF colonization affected the content of various compounds in the root exudates of maize. The root exudates of maize can inhibit the germination of the seeds of recipient plants and the growth of the seedlings of recipient plants, as well as the growth of the mycelium of the pathogenic bacteria of soil-borne diseases. AMF colonization alleviated the inhibitory effect of maize root exudates on recipient plants and improved the inhibitory effect of maize root exudates on the growth of mycelium of soil-borne disease pathogens. AMF colonization decreased the diversity of soil bacteria in maize rhizosphere, and increased the abundance of soil bacteria and the activity of soil enzymes in maize rhizosphere. The results of this study can provide a theoretical basis for the research of AMF in agricultural production and soil-borne disease prevention, as well as for maize stubble modification and intercropping mode between maize and other plants.

## Data availability statement

The data presented in the study are deposited in the “public database of figshare repository”, accession number 1050104.

## Author contributions

JH and JY participated in the experimental design; YY, SQ, JC, KC, CS, YX collected experimental materials; CJ and XY participated in the experimental analysis, and YL participated in the discussion part of the paper; JM participated in the experimental analysis, collected the experimental data, analyzed the experimental data and prepared the manuscript. All of the authors read and approved the manuscript.

## Funding

This project is supported by the National Natural Science Foundation of China (31760137) and Guangxi Natural Science Foundation (2013GXNSFAA019053).

## Conflict of interest

The authors declare that the research was conducted in the absence of any commercial or financial relationships that could be construed as a potential conflict of interest.

## Publisher’s note

All claims expressed in this article are solely those of the authors and do not necessarily represent those of their affiliated organizations, or those of the publisher, the editors and the reviewers. Any product that may be evaluated in this article, or claim that may be made by its manufacturer, is not guaranteed or endorsed by the publisher.

## Abbreviations

CE, maize colonized by Claroideoglomus etunicatum; CK, maize without AMF; FG, Fusarium graminearum; FO, Fusarium oxysporum; RS, Rhizoctonia solani.

## References

[B1] Adedokun E. O. Rather I. A. Bajpai V. K. Park Y. (2016). Biocontrol efficacy of lactobacillus fermentum yml014 against food spoilage moulds using the tomato puree model. Front. Life Sci. 9 (1), 64–68. doi: 10.1080/21553769.2015.1084951

[B2] Baetz U. Martinoia E. (2014). Root exudates: the hidden part of plant defense. Trends Plant Sci. 19 (2), 90–98. doi: 10.1016/j.tplants.2013.11.006 24332225

[B3] Bolger A. M. Lohse M. Usadel B. (2014). Trimmomatic: a flexible trimmer for illumina sequence data. Bioinformatics 30 (15), 2114–2120. doi: 10.1093/bioinformatics/btu170 24695404 PMC4103590

[B4] Bowles T. M. Acosta-Martínez V. Calderón F. Jackson L. E. (2014). Soil enzyme activities, microbial communities, and carbon and nitrogen availability in organic agroecosystems across an intensively-managed agricultural landscape. Soil Biol. Biochem. 68, 252–262. doi: 10.1016/j.soilbio.2013.10.004

[B5] Caporaso J. G. Bittinger K. Bushman F. D. Desantis T. Z. Andersen G. L. Knight R. (2010). Pynast: a flexible tool for aligning sequences to a template alignment. Bioinformatics 26 (2), 266–267. doi: 10.1093/bioinformatics/btp636 19914921 PMC2804299

[B6] Caravaca F. Ruess L. (2014). Arbuscular mycorrhizal fungi and their associated microbial community modulated by collembola grazers in host plant free substrate. Soil Biol. Biochem. 69, 25–33. doi: 10.1016/j.soilbio.2013.10.032

[B7] Chen B. Du K. Sun C. Vimalanathan A. Liang X. Li Y.. (2018). Gut bacterial and fungal communities of the domesticated silkworm (bombyx mori) and wild mulberry-feeding relatives. ISME J. 12 (9), 2252–2262. doi: 10.1038/s41396-018-0174-1 29895989 PMC6092317

[B8] Coskun D. Britto D. T. Shi W. Kronzucker H. J. (2017). How plant root exudates shape the nitrogen cycle. Trends Plant Sci. 22 (8), 661–673. doi: 10.1016/j.tplants.2017.05.004 28601419

[B9] Cosme M. Wurst S. (2013). Interactions between arbuscular mycorrhizal fungi, rhizobacteria, soil phosphorus and plant cytokinin deficiency change the root morphology, yield and quality of tobacco. Soil Biol. Biochem. 57, 436–443. doi: 10.1016/j.soilbio.2012.09.024

[B10] Denef K. Roobroeck D. Manimel Wadu M. C. W. Lootens P. Boeckx P. (2009). Microbial community composition and rhizodeposit-carbon assimilation in differently managed temperate grassland soils. Soil Biol. Biochem. 41 (1), 144–153. doi: 10.1016/j.soilbio.2008.10.008

[B11] De Vries F. T. Williams A. Stringer F. Willcocks R. Mcewing R. Langridge H.. (2019). Changes in root-exudate-induced respiration reveal a novel mechanism through which drought affects ecosystem carbon cycling. New Phytol. 224 (1), 132–145. doi: 10.1111/nph.16001 31218693 PMC6771481

[B12] Dong Q. Zhao X. Zhou D. Liu Z. Shi X. Yuan Y.. (2022). Maize and peanut intercropping improves the nitrogen accumulation and yield per plant of maize by promoting the secretion of flavonoids and abundance of bradyrhizobium in rhizosphere. Front. Plant Sci. 13. doi: 10.3389/fpls.2022.957336 PMC9386453 35991432

[B13] Edgar R. C. (2013). Uparse: highly accurate otu sequences from microbial amplicon reads. Nat. Methods 10 (10), 996–998. doi: 10.1038/nmeth.2604 23955772

[B14] Edgar R. C. Haas B. J. Clemente J. C. Quince C. Knight R. (2011). Uchime improves sensitivity and speed of chimera detection. Bioinformatics 27 (16), 2194–2200. doi: 10.1093/bioinformatics/btr381 21700674 PMC3150044

[B15] Fusconi A. (2014). Regulation of root morphogenesis in arbuscular mycorrhizae: what role do fungal exudates, phosphate, sugars and hormones play in lateral root formation? Ann. Bot-London 113 (1), 19–33. doi: 10.1093/aob/mct258 PMC3864729 24227446

[B16] Fu X. Wu X. Zhou X. Liu S. Shen Y. Wu F. (2015). Companion cropping with potato onion enhances the disease resistance of tomato against verticillium dahliae. Front. Plant Sci. 6. doi: 10.3389/fpls.2015.00726 PMC4566073 26442040

[B17] Hao W. Ren L. Ran W. Shen Q. (2010). Allelopathic effects of root exudates from watermelon and rice plants on fusarium oxysporum f.sp. Niveum. Plant Soil 336), 485–497. doi: 10.1007/s11104-010-0505-0

[B18] Jin Y. Jin J. Wang M. Wang Y. Lu W. Lu K.. (2019). Effect of plants and their root exudate on bacterial activities during rhizobacterium–plant remediation of phenol from water. Environ. Int. 127, 114–124. doi: 10.1016/j.envint.2019.03.015 30913456

[B19] Kato-Noguchi H. Sugimoto H. Yamada M. (2007). Buckwheat seedlings may inhibit other plant growth by allelopathic substances. Environ. control Biol. 45 (1), 27–32. doi: 10.2525/ecb.45.27

[B20] Klupczyńska E. A. Pawłowski T. A. (2021). Regulation of seed dormancy and germination mechanisms in a changing environment. Int. J. Mol. Sci. 22 (3), 1357. doi: 10.3390/ijms22031357 33572974 PMC7866424

[B21] Koide R. T. Mosse B. (2004). A history of research on arbuscular mycorrhiza. Mycorrhiza 14 (3), 145–163. doi: 10.1007/s00572-004-0307-4 15088135

[B22] Lanoue A. Burlat V. Schurr U. Rose U. S. (2010). Induced root-secreted phenolic compounds as a belowground plant defense. Plant Signal Behav. 5 (8), 1037–1038. doi: 10.4161/psb.5.8.12337 20699651 PMC3115191

[B23] Li J. Lin S. Zhang Q. Zhang Q. Hu W. He H. (2019). Fine-root traits of allelopathic rice at the seedling stage and their relationship with allelopathic potential. PeerJ (San Francisco CA) 7, e7006. doi: 10.7717/peerj.7006 PMC6570997 31223525

[B24] Liu N. Shao C. Sun H. Liu Z. Guan Y. Wu L.. (2020). Arbuscular mycorrhizal fungi biofertilizer improves american ginseng (panax quinquefolius l.) growth under the continuous cropping regime. Geoderma 363, 114155. doi: 10.1016/j.geoderma.2019.114155

[B25] Li X. Wei Q. Liu B. Alam M. Wang X. Shen W.. (2013). Root exudates of transgenic cotton and their effects on fusarium oxysporum. Front. bioscience (Landmark edition) 18 (2), 725. doi: 10.2741/4134 23276956

[B26] López-Ruiz B. A. Zluhan-Martínez E. Sánchez M. D. L. P. álvarez-Buylla E. R. Garay-Arroyo A. (2020). Interplay between hormones and several abiotic stress conditions on arabidopsis thaliana primary root development. Cells (Basel Switzerland) 9 (12), 2576. doi: 10.3390/cells9122576 PMC7759812 33271980

[B27] Magoc T. Salzberg S. L. (2011). Flash: fast length adjustment of short reads to improve genome assemblies. Bioinformatics 27 (21), 2957–2963. doi: 10.1093/bioinformatics/btr507 21903629 PMC3198573

[B28] Ma J. Ma Y. Wei Z. Wu J. Sun C. Yang J.. (2021). Effects of arbuscular mycorrhizal fungi symbiosis on microbial diversity and enzyme activities in the rhizosphere soil ofartemisia annua. Soil Sci. Soc. Am. J. 85 (3), 703–716. doi: 10.1002/saj2.20229

[B29] Mar Vázquez M. César S. Azcón R. Barea J. M. (2000). Interactions between arbuscular mycorrhizal fungi and other microbial inoculants (azospirillum, pseudomonas, trichoderma) and their effects on microbial population and enzyme activities in the rhizosphere of maize plants. Appl. Soil Ecol. 15 (3), 261–272. doi: 10.1016/S0929-1393.(00)00075-5

[B30] Ma J. Wang W. Yang J. Qin S. Yang Y. Sun C.. (2022). Mycorrhizal symbiosis promotes the nutrient content accumulation and affects the root exudates in maize. BMC Plant Biol. 22 (1), 64. doi: 10.1186/s12870-021-03370-2 35123400 PMC8817564

[B31] Muhammad I. Yang L. Ahmad S. Zeeshan M. Farooq S. Ali I.. (2022). Irrigation and nitrogen fertilization alter soil bacterial communities, soil enzyme activities, and nutrient availability in maize crop. Front. Microbiol. 13. doi: 10.3389/fmicb.2022.833758 PMC8851207 35185852

[B32] Nagahashi G. Douds D. (2007). Separated components of root exudate and cytosol stimulate different morphologically identifiable types of branching responses by arbuscular mycorrhizal fungi. Mycological Res. 111 (4), 487–492. doi: 10.1016/j.mycres.2007.02.007 17544057

[B33] Nardi S. Sessi E. Pizzeghello D. Sturaro A. Rella R. Parvoli G. (2002). Biological activity of soil organic matter mobilized by root exudates. Chemosphere 46 (7), 1075–1081. doi: 10.1016/s0045-6535(01)00160-6 11999770

[B34] Ntushelo K. Ledwaba L. K. Rauwane M. E. Adebo O. A. Njobeh P. B. (2019). The mode of action of bacillus species against fusarium graminearum , tools for investigation, and future prospects. Toxins 11 (10), 606. doi: 10.3390/toxins11100606 31635255 PMC6832908

[B35] Oghenekaro A. O. Oviedo-Ludena M. A. Serajazari M. Wang X. Henriquez M. A. Wenner N. G.. (2021). Population genetic structure and chemotype diversity of fusarium graminearum populations from wheat in canada and north eastern united states. Toxins (Basel) 13 (3), 180. doi: 10.3390/toxins13030180 33804426 PMC7999200

[B36] Pantigoso H. A. Yuan J. He Y. Guo Q. Vollmer C. Vivanco J. M.. (2020). Role of root exudates on assimilation of phosphorus in young and old arabidopsis thaliana plants. PloS One 15 (6), e234216. doi: 10.1371/journal.pone.0234216 PMC7269232 32492072

[B37] Peng Q. Wu M. Zhang Z. Su R. He H. Zhang X. (2020). The interaction of arbuscular mycorrhizal fungi and phosphorus inputs on selenium uptake by alfalfa (medicago sativa l.) and selenium fraction transformation in soil. Front. Plant Sci. 11. doi: 10.3389/fpls.2020.00966 PMC7333729 32676094

[B38] Pereira S. I. A. Moreira H. Argyras K. Castro P. M. L. Marques A. P. G. C. (2016). Promotion of sunflower growth under saline water irrigation by the inoculation of beneficial microorganisms. Appl. Soil Ecol. 105, 36–47. doi: 10.1016/j.apsoil.2016.03.015

[B39] Quast C. Pruesse E. Yilmaz P. Gerken J. Schweer T. Yarza P.. (2012). The silva ribosomal rna gene database project: improved data processing and web-based tools. Nucleic Acids Res. 41 (D1), D590–D596. doi: 10.1093/nar/gks1219 23193283 PMC3531112

[B40] Rabea E. I. Badawy M. E. I. Steurbaut W. Stevens C. V. (2009). *In vitro* assessment of n- (benzyl)chitosan derivatives against some plant pathogenic bacteria and fungi. Eur. Polym J. 45 (1), 237–245. doi: 10.1016/j.eurpolymj.2008.10.021

[B41] Sasse J. Martinoia E. Northen T. (2018). Feed your friends: do plant exudates shape the root microbiome? Trends Plant Sci. 23 (1), 25–41. doi: 10.1016/j.tplants.2017.09.003 29050989

[B42] Sela A. Piskurewicz U. Megies C. Mene-Saffrane L. Finazzi G. Lopez-Molina L. (2020). Embryonic photosynthesis affects post-germination plant growth. Plant Physiol. 182 (4), 2166–2181. doi: 10.1104/pp.20.00043 32060052 PMC7140907

[B43] Shamya Arokia Rajan M. Thriunavukkarasu R. Joseph J. Aruni W. (2020). Effect of seaweed on seed germination and biochemical constituents of capsicum annuum. Biocatalysis Agric. Biotechnol. 29, 101761. doi: 10.1016/j.bcab.2020.101761

[B44] Shukla A. Dehariya K. Vyas D. Jha A. (2015). Interactions between arbuscular mycorrhizae and fusarium oxysporum f. sp. ciceris: effects on fungal development, seedling growth and wilt disease suppression in cicer arietinum l. Archiv für Phytopathologie und Pflanzenschutz 48 (3), 240–252. doi: 10.1080/03235408.2014.884831

[B45] Singh S. Singh U. B. Malviya D. Paul S. Sahu P. K. Trivedi M.. (2020). Seed biopriming with microbial inoculant triggers local and systemic defense responses against rhizoctonia solani causing banded leaf and sheath blight in maize (zea mays l.). Int. J. Environ. Res. Public Health 17 (4), 1396. doi: 10.3390/ijerph17041396 32098185 PMC7068308

[B46] Song Q. Cheng S. Chen Z. Nie G. Xu F. Zhang J.. (2019). Comparative transcriptome analysis revealing the potential mechanism of seed germination stimulated by exogenous gibberellin in fraxinus hupehensis. BMC Plant Biol. 19 (1), 199. doi: 10.1186/s12870-019-1801-3 31092208 PMC6521437

[B47] Stamou G. P. Konstadinou S. Monokrousos N. Mastrogianni A. Orfanoudakis M. Hassiotis C.. (2017). The effects of arbuscular mycorrhizal fungi and essential oil on soil microbial community and n-related enzymes during the fungal early colonization phase. AIMS Microbiol. 3 (4), 938–959. doi: 10.3934/microbiol.2017.4.938 31294199 PMC6604959

[B48] Staszel K. Lasota J. Blonska E. (2022). Effect of drought on root exudates from quercus petraea and enzymatic vigor of soil. Sci. Rep. 12 (1), 7635. doi: 10.1038/s41598-022-11754-z 35538167 PMC9090927

[B49] Suarez-Fernandez M. Marhuenda-Egea F. C. Lopez-Moya F. Arnao M. B. Cabrera-Escribano F. Nueda M. J.. (2020). Chitosan induces plant hormones and defenses in tomato root exudates. Front. Plant Sci. 11. doi: 10.3389/fpls.2020.572087 PMC7672008 33250907

[B50] Sun Z. K. He W. M. (2019). Autotoxicity of root exudates varies with species identity and soil phosphorus. Ecotoxicology 28 (4), 429–434. doi: 10.1007/s10646-019-02035-z 30904977

[B51] Tian B. Pei Y. Huang W. Ding J. Siemann E. (2021). Increasing flavonoid concentrations in root exudates enhance associations between arbuscular mycorrhizal fungi and an invasive plant. Isme J. 15 (7), 1919–1930. doi: 10.1038/s41396-021-00894-1 33568790 PMC8245413

[B52] Turk M. A. Tawaha A. M. (2003). Allelopathic effect of black mustard (brassica nigra l.) on germination and growth of wild oat (avena fatua l.). Crop Prot 22 (4), 673–677. doi: 10.1016/S0261-2194(02)00241-7

[B53] Wang Q. Garrity G. M. Tiedje J. M. Cole J. R. (2007). Naiüve bayesian classifier for rapid assignment of rrna sequences into the new bacterial taxonomy. Appl. Environ. Microb. 73 (16), 5261–5267. doi: 10.1128/AEM.00062-07 PMC1950982 17586664

[B54] Yang L. Muhammad I. Chi Y. X. Wang D. Zhou X. B. (2022). Straw return and nitrogen fertilization to maize regulate soil properties, microbial community, and enzyme activities under a dual cropping system. Front. Microbiol. 13. doi: 10.3389/fmicb.2022.823963 PMC8965350 35369510

[B55] Yizhu L. Imtiaz M. Ditta A. Rizwan M. S. Ashraf M. Mehmood S.. (2020). Response of growth, antioxidant enzymes and root exudates production towards as stress in pteris vittata and in astragalus sinicus colonized by arbuscular mycorrhizal fungi. Environ. Sci. pollut. R 27 (2), 2340–2352. doi: 10.1007/s11356-019-06785-5 31776909

[B56] Zarea M. J. Karimi N. Goltapeh E. M. Ghalavand A. (2011). Effect of cropping systems and arbuscular mycorrhizal fungi on soil microbial activity and root nodule nitrogenase. J. Saudi Soc. Agric. Sci. 10 (2), 109–120. doi: 10.1016/j.jssas.2011.04.003

[B57] Zhang Y. He J. Jia L. J. Yuan T. L. Zhang D. Guo Y.. (2016). Cellular tracking and gene profiling of fusarium graminearum during maize stalk rot disease development elucidates its strategies in confronting phosphorus limitation in the host apoplast. PloS Pathog. 12 (3), e1005485. doi: 10.1371/journal.ppat.1005485 26974960 PMC4790934

[B58] Zhang G. Raza W. Wang X. Ran W. Shen Q. (2012). Systemic modification of cotton root exudates induced by arbuscular mycorrhizal fungi and bacillus vallismortis hj-5 and their effects on verticillium wilt disease. Appl. Soil Ecol. 61, 85–91. doi: 10.1016/j.apsoil.2012.02.003

[B59] Zhang Y. Zheng Y. Xia P. Xun L. Liang Z. (2019). Impact of continuous panax notoginseng plantation on soil microbial and biochemical properties. Sci. Rep. 9 (1), 13205. doi: 10.1038/s41598-019-49625-9 31519939 PMC6744506

[B60] Zou P. Lu X. Zhao H. Yuan Y. Meng L. Zhang C.. (2019). Polysaccharides derived from the brown algae lessonia nigrescens enhance salt stress tolerance to wheat seedlings by enhancing the antioxidant system and modulating intracellular ion concentration. Front. Plant Sci. 10. doi: 10.3389/fpls.2019.00048 PMC6365471 30766543

[B61] Zubek S. Stefanowicz A. M. Błaszkowski J. Niklińska M. Seidler-Bożykowska K. (2012). Arbuscular mycorrhizal fungi and soil microbial communities under contrasting fertilization of three medicinal plants. Appl. Soil Ecol. 59, 106–115. doi: 10.1016/j.apsoil.2012.04.008

